# Frameshift Mutation Confers Function as Virulence Factor to Leucine-Rich Repeat Protein from *Acidovorax avenae*

**DOI:** 10.3389/fpls.2016.01988

**Published:** 2017-01-04

**Authors:** Machiko Kondo, Hiroyuki Hirai, Takehito Furukawa, Yuki Yoshida, Aika Suzuki, Takemasa Kawaguchi, Fang-Sik Che

**Affiliations:** Graduate School of Bioscience, Plant Molecular Physiology, Nagahama Institute of Bio-Science and TechnologyNagahama, Japan

**Keywords:** effector, virulent, leucine rich repeat, rice, finger millet, brown stripe, *hrp*, T3SS

## Abstract

Many plant pathogens inject type III (T3SS) effectors into host cells to suppress host immunity and promote successful infection. The bacterial pathogen *Acidovorax avenae* causes brown stripe symptom in many species of monocotyledonous plants; however, individual strains of each pathogen infect only one host species. T3SS-deleted mutants of *A. avenae* K1 (virulent to rice) or N1141 (virulent to finger millet) caused no symptom in each host plant, suggesting that T3SS effectors are involved in the symptom formation. To identify T3SS effectors as virulence factors, we performed whole-genome and predictive analyses. Although the nucleotide sequence of the novel leucine-rich repeat protein (*Lrp*) gene of N1141 had high sequence identity with K1 Lrp, the amino acid sequences of the encoded proteins were quite different due to a 1-bp insertion within the K1 *Lrp* gene. An Lrp-deleted K1 strain (*KΔLrp*) did not cause brown stripe symptom in rice (host plant for K1); by contrast, the analogous mutation in N1141 (*NΔLrp*) did not interfere with infection of finger millet. In addition, *NΔLrp* retained the ability to induce effector-triggered immunity (ETI), including hypersensitive response cell death and expression of ETI-related genes. These data indicated that K1 Lrp functions as a virulence factor in rice, whereas N1141 Lrp does not play a similar role in finger millet. Yeast two-hybrid screening revealed that K1 Lrp interacts with oryzain α, a pathogenesis-related protein of the cysteine protease family, whereas N1141 Lrp, which contains LRR domains, does not. This specific interaction between K1 Lrp and oryzain α was confirmed by Bimolecular fluorescence complementation assay in rice cells. Thus, K1 Lrp protein may have acquired its function as virulence factor in rice due to a frameshift mutation.

## Introduction

Plants are constantly being challenged by potentially disease-causing microorganisms. In order to successfully infect host plants and establish disease, bacterial pathogens require weaponry to facilitate infiltration, host colonization, and uptake of nutrients for growth and reproduction. On the other hand, plants have developed a sophisticated multi-layered immune system to defend themselves against invading pathogens (Jones and Dangl, 2006). The first layer of the plant immune system is pattern-triggered immunity (PTI), which is triggered by specific recognition of conserved PAMPs (pathogen-associated molecular patterns) or MAMPs (microbe-associated molecular patterns) by pattern recognition receptors (PRRs) at the plasma membrane and the induction of immune signaling (Boller and Felix, 2009; Monaghan and Zipfel, 2012). Examples of PAMPs or MAMPs identified to date include β-glucan (Klarzynski et al., 2000), polysaccharide chitin (Kaku et al., 2006), ergosterol (Laquitaine et al., 2006), flagellin (Felix et al., 1999; Che et al., 2000), lipopolysaccharide (LPS) (Silipo et al., 2005), translation elongation factor EF-Tu (Kunze et al., 2004; Furukawa et al., 2014), and elicitin (Baillieul et al., 2003). PTI response induced by PAMPs or MAMPs was contained generation of reactive oxygen species, callose deposition, and expression of several PTI-related genes (Boller and Felix, 2009).

Another layer of the plant immune system involves molecular recognition of effector proteins secreted from bacterial type III secretion system (T3SS), which injects multiple proteins into a plant cell. These bacterial effector proteins may have evolved to suppress PTI to achieve successful infection (Alfano and Collmer, 2004; Chisholm et al., 2006). Plants, in turn, evolved resistance proteins that can directly or indirectly recognize the effector proteins. This recognition response, associated with the long-standing gene-for-gene theory, is now known as effector-triggered immunity (ETI). ETI is frequently associated with development of the hypersensitive response (HR), a form of programmed cell death localized at the infection site, which prevents the spread of the pathogen inside the plant (Mur et al., 2008). In many plant pathogenic bacteria, the T3SS apparatus and related proteins are encoded by genes located in *hrp* (hypersensitive response and pathogenicity) gene clusters. In *Xanthomonas campestris* pv. *vesicatoria*, the causal agent of bacterial spot disease in pepper and tomato plants, the T3SS is encoded by at least six loci, clustered in a 23-kb *hrp* gene cluster. The expression of the *hrp* operons is activated in plants by the products of two regulatory genes, *hrpG* and *hrpX*. HrpG, a member of the OmpR family of two-component response regulators, controls the *hrpG* regulon, which contains genes for type III effector proteins; in most cases, HrpG exerts its regulatory effects via HrpX (Noël et al., 2001).

Although animal pathogenic bacteria secrete only a limited number of effectors into host animal cells, plant pathogenic bacteria, such as *Pseudomonas syringae* and *X. campestris* secrete 20–40 effectors during infection (Macho, 2016). The whole-genome sequence of *P. syringae* revealed that the super-repertoire of effectors in the complex comprises 57 families (Lindeberg et al., 2012). The major role that has been assigned to these effectors is the suppression of plant immunity, which would otherwise prevent bacterial colonization and growth. By their collective action, effectors alter plant physiology in susceptible hosts, thereby sustaining pathogen growth. Many types of bacterial effector proteins target cellular functions of host plants that are not directly related to immunity. For example, HopX1 from *P. syringae* regulate JA signaling through the degradation of JAZ protein (Gimenez-Ibanez et al., 2014). PthXo1 and AvrXa7, the *X. oryzae* transcription activator-like effectors (TALEs), induce expression of several genes encoding members of the SWEET family of sugar transporters, promoting sugar eﬄux in the affected plant cells (Streubel et al., 2013; Macho, 2016). AvrRpt2, HopQ1, and AvrPtoB from *P. syringae*, can sensitize the auxin, cytokinin, and abscisic acid signaling pathways in host plant cells, respectively, and AvrBS3 and AvrXccC from *X. campestris* can induce auxin and abscisic acid signaling, respectively. The *P. syringae* T3SS effector HopZ1 targets the isoflavone biosynthesis enzyme GmHID1, and suppresses the isoflavone biosynthetic pathway to promote bacterial infection (Zhou et al., 2011). Moreover, WtsE from *Pantoea stewartii* upregulates the shikimate and phenylpropanoid pathways, promoting pathogen virulence (Asselin et al., 2015).

T3SS effector proteins often promote disease by suppressing immune signaling or manipulating host functions. In addition, the T3SS effector proteins also induce ETI. The dual role of T3SS effector proteins often depends on the host genotype. For example, the T3SS effector AvrBs3 from *X. campestris* elicits ETI resistance in tomato plants possessing *Bs3* genes, but functions as a virulence factor in tomato plants lacking these genes (Marois et al., 2002). *P. syringae* carrying the T3SS effectors AvrRpt2, AvrRpm1, and AvePphB causes disease symptoms in host plants lacking the resistance protein RPS2, but clearly induced ETI associated with HR in host plants possessing this protein. RPS2 represents a family of proteins containing nucleotide-binding (NB) site and leucine-rich repeat (LRR) domains. Activation of the NB-LRR can be triggered by direct interaction with the effector or by monitoring host proteins that are modified by the effector (guard model). In the guard model, the “guarded” protein is both the virulence target of the effector protein in hosts lacking the cognate resistance protein NB-LRR, and part of the defense mechanism in hosts carrying that protein (Schreiber et al., 2016). The decoy model posits that host proteins with no actual role in virulence evolve to resemble virulence targets, competing for binding with bacterial effectors and slowing the progress of infection (Lindeberg et al., 2012).

Rice (*Oryza sativa* L.) is one of the most important crops worldwide and a model plant for molecular studies in other monocotyledonous species. In a serious disease that leads to a decrease in the yield of rice, the brown stripe disease caused by *Acidovorax avenae* is hardly controllable (Kadota et al., 1996). *A. avenae* is a Gram-negative bacterium with a wide host range among monocotyledonous plants; however, individual strains of this pathogen each infect only one or a few host species. For example, the K1 strain isolated from rice can only infect rice plants (virulent), whereas the N1141 strain isolated from finger millet cannot infect rice, even if forcibly inoculated (avirulent). We reported that the rice-avirulent N1141 strain causes several immune responses, including HR cell death, H_2_O_2_ generation, and the up-regulation of defense genes, whereas the rice-virulent K1 strain does not induce these immune responses (Che et al., 1999). The induction of all known immune responses indicates that the host-species specificity of *A. avenae* is involved in immune induction. We recently reported that flagellin and EF-Tu of *A. avenae* N1141 function as PAMPs and induce PTI responses. Moreover, neither flg22, which is sufficient to produce the flagellin response in *Arabidopsis*, nor elf18, which contains an epitope that induces PTI in *Arabidopsis*, elicit PTI responses in rice. However, the C-terminal CD2–1 fragments of flagellin and the middle-domain EFa50 fragments of EF-Tu induce PTI responses in rice cells (Furukawa et al., 2014; Katsuragi et al., 2015). These findings indicated that flagellin and EF-Tu recognition systems of rice differ from those of *Arabidopsis* (Wang et al., 2015). Thus, although we have many knowledges about rice PTI induced by *A. avenae*, the detailed mechanism underlying the host specificity of *A. avenae* remains incompletely understood.

We previously reported that *A. avenae* rice-avirulent N1141 possesses an *hrp* cluster containing genes encoding the T3SS apparatus. A T3SS-deleted N1141 mutant (*NΔT3SS*) does not cause ETI responses, suggesting that *A. avenae* has T3SS effector proteins that can induce the ETI (Kondo et al., 2012). Here, we show that the rice-virulent K1 strain also has the *hrp* cluster, and that the *hrp* genes of K1 are involved in generation of brown stripe symptoms in rice. We also identified a novel leucine-rich domain protein (Lrp) from *A. avenae* N1141 as a candidate T3SS effector protein. Although *Lrp* genes are present in the K1 genome, there is limited amino-acid sequence similarity between N1141 and K1 Lrp due to a 1-bp insertion within the *Lrp* gene. Lrp-deleted K1 (*KΔLrp*) strain did not cause brown stripe symptoms in rice (the host plant of K1), whereas symptoms were not altered when an *Lrp*-deleted N1141 (*NΔLrp*) strain was used to infect finger millet (the host plant of N1141). These data indicated that K1 Lrp functions as a virulence factor in rice, whereas N1141 Lrp does not. Yeast two-hybrid screening revealed that K1 Lrp interacts with oryzain α, a pathogenesis-related protein, whereas N1141 Lrp containing an LRR domain does not. These findings will provide insight into the host specificity of *A. avenae.*

## Materials and Methods

### Plants and Bacteria

Rice (*O. sativa* L. cv. Nipponbare) was grown for 3–4 weeks in a natural-light phytotron at 30/25°C (day/night). One day before inoculation, the plants were placed in a growth chamber with a 16 h day (200 μE m^-2^ s^-1^ at 30°C) and 8 h night (25°C) cycle and 65% relative humidity. Finger millet (*Eleusine coracana*) was grown for 3–4 weeks in a growth chamber under 16 h day (200 μE m^-2^ s^-1^ at 30°C) and 8 h night (25°C) cycle at 65% relative humidity. Suspension cultures of rice cells (line Oc) were grown at 30°C under light irradiation. The cells were diluted in fresh medium every 7 days, and experiments were performed 4 days after transfer.

*Acidovorax avenae* strain N1141 (MAFF 301141) isolated from finger millet and strain K1 (MAFF 301755) isolated from rice were used as previously described (Kadota et al., 1996; Che et al., 2000).

### Inoculation Test

For the inoculation of *A. avenae*, the bacteria were suspended in sterilized distilled water (2 × 10^9^ cfu/ml). One microliter (10^6^ cfu) drop of the bacterial suspension medium was formed on the end of a needle and then the sheath of 3–4 weeks-old seedlings were pricked at a point 3 cm above the soil level. Control plants were mock-inoculated with sterilized water. Inoculated seedlings were maintained in a growth chamber under the same conditions. Seven days after inoculation, the pathogenicity of each strain was determined by assessing the brown stripe development around the inoculation site.

For determining the growth of *A. avenae*, seedlings were inoculated in the same manner. The growth of each strain was assessed in inoculated plants up to 4 days after inoculation. Five sets of randomly selected rice plants were harvested, rinsed thoroughly in sterile water, and homogenized in distilled water. Dilutions of the homogenate were plated onto Pseudomonas F agar. After incubation for 24 h at 30°C, the number of colony-forming units (cfu) was determined.

### Detection of HR Cell Death

Hypersensitive response cell death in cultured rice cells was detected by Evans blue staining as described previously (Che et al., 1999). Cultured rice cells were inoculated with bacteria (10^8^ cfu/ml), and incubated at 30°C. In each time point, the cultured rice cells were moved in 24-well tissue culture plates and stained with 0.05% Evans blue. After washing to remove excess dye, The Evans blue dye was extracted by extraction buffer (50% methanol and 1% SDS) for 12 h at room temperature. The extracted dye was measured at absorbance at 595 nm.

### Quantitative Real-Time RT-PCR

Total RNA was isolated from cultured rice cells using an RNeasy Plant Mini Kit (Qiagen, Hilden, Germany) with DNase digestion following to the manufacture instructions. Quantitative real-time RT-PCR was performed on an Opticon2 (Bio-Rad, Hercules, CA, USA) using a QuantiTect SYBR Green RT-PCR Kit (Qiagen) with the following *PAL* gene-specific primers (accession no: X16099): *PAL* gene-F and *PAL* gene-R, and *LOX* gene-specific primers (accession no: D14000): *LOX* gene-F and *LOX* gene-R (Supplementary Table S1). The sizes of the PCR products were examined to confirm that only mRNA was amplified in all the qRT-PCR experiments. The fluorescence data produced sigmoidal amplification plots in which the number of cycles was plotted against fluorescence. Quantification of each mRNA was calculated from threshold points located in the log-linear range of the RT-PCR. Standard samples of known template amounts were used to quantify the PCR products. The normalization was performed based on *Act-1.*

### Generation of *T3SS* and *Lrp* Deletion Mutants

The T3SS-deleted N1141 mutant was used as the previous reports (Kondo et al., 2012). The T3SS-deleted K1 mutant was produced for this research. To generate a T3SS-deleted K1 mutant, upstream region of *hrcV* and downstream region of *hrcQ* were PCR amplified using two sets of specific primers (K1-29231-F-up_spe and K1-30297-R-up for the upstream region, and K1-32115-F-down and K1-33096-R-down_spe for the downstream region), respectively. Each amplified PCR products was diluted and mixed equally. The mixed PCR products were re-amplified using the K1-29231-F-up_spe and K1-33096-R-down_spe primers. The 2-kbp PCR products were cloned into pGEM-T vector (Promega), and the resulting plasmid was digested with *Spe*I. The digested fragments were cloned into pK18*mobsacBTMP*, and the resulting plasmid was designated pK18*mobsacBTMP-KΔT3SS*. The pK18*mobsacBTMP-NΔT3SS* and pK18*mobsacBTMP-KΔT3SS* were electro-transformed into *A. avenae* N1141 and K1, respectively. The bacterial cells were plated on LB agar plates (containing 20 μg/ml the kanamycin) and incubated for 48 h at 30°C. For second crossing-over event, the colonies were incubated in sucrose selection media (Pseudomonas F liquid medium containing 26% sucrose) for 72 h at 30°C. These mutants were named *NΔT3SS* and *KΔT3SS.*

The *Lrp-*deleted N1141 mutant (*NΔLrp*) was used same strain produced in our previous reports [Kondo et al., 2012, accession no: BAE80273.1 (N1141Lrp)]. The *Lrp-*deleted K1 mutant was produced for this research [accession no: BAE80241.1 (K1Lrp)]. To generate the *Lrp-*deleted K1 mutants, upstream and downstream regions of *Lrp* were PCR amplified using two sets of specific primers (KLRP-UP-F and KLRP-UP-R for the upstream region of K1 *Lrp*, and KLRP-DOWN-F and KLRP-DOWN-R for the downstream region of K1 *Lrp*), respectively. Each amplified PCR products was diluted and mixed. The mixed PCR products were re-amplified using the KLRP-UP-F and KLRP-DOWN-R primers. The 2-kb DNA fragments were cloned into pGEM-T vector and the plasmid was digested with *Spe*I. The digested DNA fragments were ligated to pK18*mobsacBTMP* and the plasmid was named pK18*mobsacBTMP-KΔLrp*. The pK18*mobsacBTMP-KΔLrp* was transformed into K1 by electroporation. The transformed bacterial cells were plated on LB agar plates (containing 20 mg/ml of kanamycin) and incubated for 48 h at 30°C. The resulting colonies were inoculated into Pseudomonas F liquid medium (containing 26% sucrose) and incubated for 72 h at 30°C to excise the plasmid by a second crossing-over event. The resulting bacteria were designated *KΔLrp.*

### Genome Sequencing

Genomic DNA sequencing of *A. avenae* K1 and N1141 strains were performed by next-generation sequencing technology using illumina and PacBio RS. Sequence assembly was performed with HGAP and Velvet. A further sequence using primer walking were generated during the gap-closure and finishing phase. The sequence assembly was performed with ATSQ.

Annotation was performed with MiGAP (Microbial Genome Annotation Pipeline). Mapping and comparison were visualized with Artemis DNA plotter and Artemis Comparison Tool (ACT). Comparison between amino acid sequences of K1 and N1141 was performed with BLAST Global Align.

### Prediction of T3SS Effectors

Multi-FASTA files were generated by StringFormatter (included in Genome Matcher system). EffectiveT3 prediction was performed using each Multi-FASTA files. The threshold was >0.9999 (secreted) in EffectiveT3 prediction.

### Yeast Two-Hybrid

Full-length K1 or N1141 *Lrp* coding regions were PCR amplified from N1141 genomic DNA or K1 genomic DNA with a set of specific oligonucleotide primers [K1 Lrp (BKT7-EcoRI)-F and K1 Lrp (BKT7-BamHI)-R, N1141 Lrp (BKT7-EcoRI)-F and N1141 Lrp (BKT7-EcoRI)-R], respectively. The amplified products (2.2 kbp or 1.6 kbp) were ligated into pGEM-T vector. The resulting plasmid, pGEM/*K1 Lrp*, was digested with *EcoR*I and *BamH*I. Other resulting plasmid, pGEM/*N1141 Lrp*, was digested with *EcoR*I. DNA fragment of *K1 Lrp* or *N1141 Lrp* were isolated, and ligated into the *EcoR*I and *BamH*I or *EcoR*I only digestion sites within the pGBKT7 vector, respectively. The resulting plasmids were designated pGBKT7/*K1 Lrp* and pGBKT7/*N1141 Lrp* as bait vector. Full-length *Oryzain α* cording region was PCR amplified from rice full-length cDNA clone (Genebank Project, NARO, J013002H09) with a set of specific oligonucleotide primers (Oryzain(EcoRI)-F and Oryzain(EcoRI)-R). The amplified product (1.8 kbp) was cloned into the pCR-Blunt vector (Invitrogen). The resulting plasmid, pBlunt/*Oryzain*, was digested with *EcoR*I. DNA fragment of *Oryzain α* was isolated and ligated into *EcoR*I digestion sites within the pGADT7 vector. The resulting plasmid was designated pGADT7/*Oryzain* as prey vector. N-terminus-truncated fragments of *Oryzain α* coding regions were PCR amplified from pGADT7/*Oryzain* prey vector with a set of specific oligonucleotide primers [Oryzain 236 (EcoRI)-F and Oryzain (EcoRI)-R, Oryzain 340 (EcoRI)-F and Oryzain (EcoRI)-R], respectively. The amplified products (0.67 kbp or 0.36 kbp) were ligated into pCR-Blunt vector. The resulting plasmids, pBlunt/*Oryzain* 236-459 and pBlunt/*Oryzain* 340-459, were digested with *EcoR*I. DNA fragments of N-terminus-truncated *Oryzain α* were isolated, and ligated into the *EcoR*I digestion sites within the pGADT7 vector, respectively. The resulting plasmids were designated pGADT7/*Oryzain* 236-459 and pGADT7/*Oryzain* 340-459 as prey vector. The plasmids pGBKT7/*K1 Lrp* or pGBKT7/*N1141* Lrp, and pGADT7/*Oryzain* or pGADT7/*Oryzain* 236-459 and pGADT7/*Oryzain* 340-459 were cotransformed into *S*. *cerevisiae* strain AH109. Plasmids pGBKT7/*p53* and pGADT7/*T* served a positive control, and plasmids pGBKT7/*Lam* and pGADT7/*T* were used as negative control. Transformants were grown at 30°C for 72 h on synthetic complete (SC) medium lacking Leu and Trp and then transferred to several selection media (lacking His, Leu and Trp, lacking His, Leu and Trp and containing X-α-gal, lacking Ade, His, Leu and Trp). Three independent experiments were performed to confirm the result.

### Bimolecular Fluorescence Complementation

Full-length K1 or N1141 *Lrp* coding regions were PCR amplified from K1 or N1141 genomic DNAs with a set of specific primers (K1_lrp_pENTER_cacc_F and K1_lrp_pENTER_without_stop_R, N1141_lrp_pENTER_cacc_F and N1141_lrp_pENTER_without_stop_R), respectively. Full-length *oryzain α* (accession no: DQ222400.1) coding region was PCR amplified from a rice cDNA library with the specific primers (oryzain_pENTER_cacc_F and oryzain_pENTER_without_stop_R). *GUS* (the β-glucuronidase encoding gene) was also amplified from pBI221 with specific primers (GUS-pENTR_F and GUS-pENTR_R). The resulting PCR products were cloned into pENTR-D-TOPO (Invitrogen, Carlsbad, CA, USA). These plasmids were converted to the gateway-compatible destination vectors (GW-Vn/pBI221 or GW-Vc/pBI221, Kamimura et al., 2014) by the Gateway vector conversion system. The resulting plasmids were designated KLrp-VN-pBI221, NLrp-VN-pBI221, GUS-VN-pBI221, Oryzain-VC-pBI221, and GUS-VC-pBI221 as bimolecular fluorescence complementation (BiFC) vectors.

After 4 days of culture, cultured rice cells were harvested and plated on R2O agar at 30°C for 3 h. The BiFC vectors and pAHC17-DsRed (Kaneda et al., 2009) were co-introduced by particle gun bombardment according to the manufacturer’s protocol (Bio-Rad). After 8 h incubation at 30°C, BiFC fluorescence was observed using the confocal laser scanning microscope (FV1000, OLYMPUS, Tokyo, Japan).

## Results

### Roles of N1141 and K1 T3SS in Lesion Formation

Many plant pathogenic bacteria use T3SS to inject effector proteins into plant cells. These effector proteins are considered to be virulence factors in host cells, as well as factors that induce immune responses, such as HR in non-host cells. Mutations in *hrp* genes generally abolish pathogenicity in susceptible host plants and the induction of immune responses (including HR cell death) in resistant non-host plants. We previously reported that the *A. avenae* N1141 strain possesses an *hrp* cluster containing genes encoding T3SS constituents (Kondo et al., 2012). Therefore, we constructed T3SS-deleted mutants of N1141 and K1 to investigate whether effector proteins in N1141 or K1 strains function as virulence or avirulence factors.

Among the *hrp* genes, *hrcV, hrcQ, hrcR*, and *hrcS* encode components of the T3SS apparatus, and the sequences of these genes are conserved within Gram-negative bacteria. Therefore, we generated *hrcV*–*hrcQ* deletion mutants of N1141 and K1 by homologous recombination. For this purpose, the *hrcV* upstream and *hrcQ* downstream region from N1141 and K1 were ligated into pK18*mobsacBTMP*. Isogenic *hrcV*–*hrcQ* deletion mutants were made using these plasmids, and the resultant mutants were designated *NΔT3SS* and *KΔT3SS*. In liquid medium, the mutants grew at the same rates as wild-type N1141 and K1 (Supplementary Figure S1).

**FIGURE 1:**
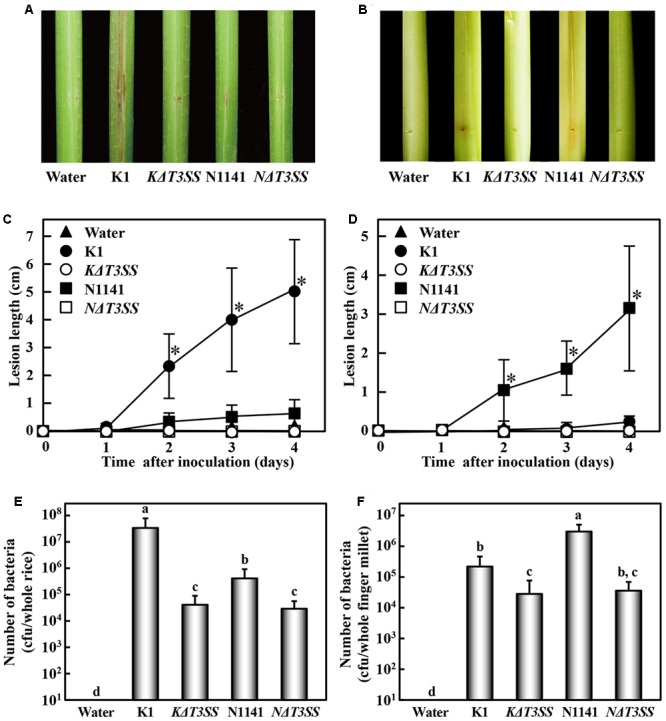
**Virulence test of T3SS-deleted mutants in rice and finger millet. (A)** Phenotype of brown stripe symptoms in rice 7 days after inoculation. **(B)** Phenotype of brown stripe symptoms in finger millet 7 days after inoculation. Bacterial strains (1 × 10^6^ cfu) were inoculated into 30-day-old rice seedlings using the single-needle method. **(C)** Progression of average brown stripe symptoms in rice. **(D)** Progression of average brown stripe symptoms in finger millet. Lesion lengths are represented as means and standard deviations calculated from three data points. Solid triangle, water (control); solid circles, K1 strain; open circles, *KΔT3SS*, solid squares, N1141; open squares, *NΔT3SS*. **(E)** Number of bacterial cells in whole rice plants 4 days after inoculation. Bars indicate standard deviation of nine experiments. **(F)** Number of bacterial cells in whole finger millet plants 4 days after inoculation. Bars indicate standard deviation of nine experiments. Asterisks in **(C)** and **(D)** indicate significant differences between K1 and others **(C)**, or N1141 and others **(D)** according to *post hoc* ANOVA Tukey-Kramer test (*P* < 0.01). Small-case letters above bars in **(E)** and **(F)** indicate significant differences according to *post hoc* ANOVA Tukey-Kramer test (*P* < 0.01).

To evaluate the ability to form lesions, K1, *KΔT3SS*, N1141, and *NΔT3SS* were inoculated into rice (host plant for K1) and finger millet (host plant for N1141). When rice plants were inoculated with strain K1 and maintained at 30°C, brown stripe lesions were observed around the inoculation points on the rice sheath 7 days after inoculation (**Figures 1A,C**). No remarkable brown stripe symptoms were observed in rice inoculated with *KΔT3SS*, N1141, or *NΔT3SS* (**Figures 1A,C**). By contrast, clear brown stripe symptoms were observed in N1141-inoculated finger millet 7 days after inoculation, whereas K1, *KΔT3SS*, and *NΔT3SS* caused no symptoms in millet (**Figures 1B,D**).

To determine whether the lack of symptoms was associated with diminished pathogen growth in rice, we monitored the numbers of bacteria in inoculated rice and finger millet. In rice inoculated with each bacterial strain (1 × 10^6^ cfu), the number of cells of strain K1 reached 3 × 10^7^ cfu/plant 4 days after inoculation. By contrast, the number of cells of *KΔT3SS*, N1141, and *NΔT3SS* decreased 4 days after inoculation (**Figure 1E**). When N1141 was inoculated into finger millet, the number of cells increased 4 days after inoculation (**Figure 1F**), whereas the cell numbers of K1, *KΔT3SS*, and *NΔT3SS* decreased (**Figure 1F**). In all experiments, mock-inoculated plants (negative controls) remained healthy, and no bacterial contamination was detected. These results indicate that the lack of brown stripe symptoms is associated with diminished pathogen growth in rice and finger millet.

### Roles of K1 and N1141 T3SSs on ETI Induction

**FIGURE 2:**
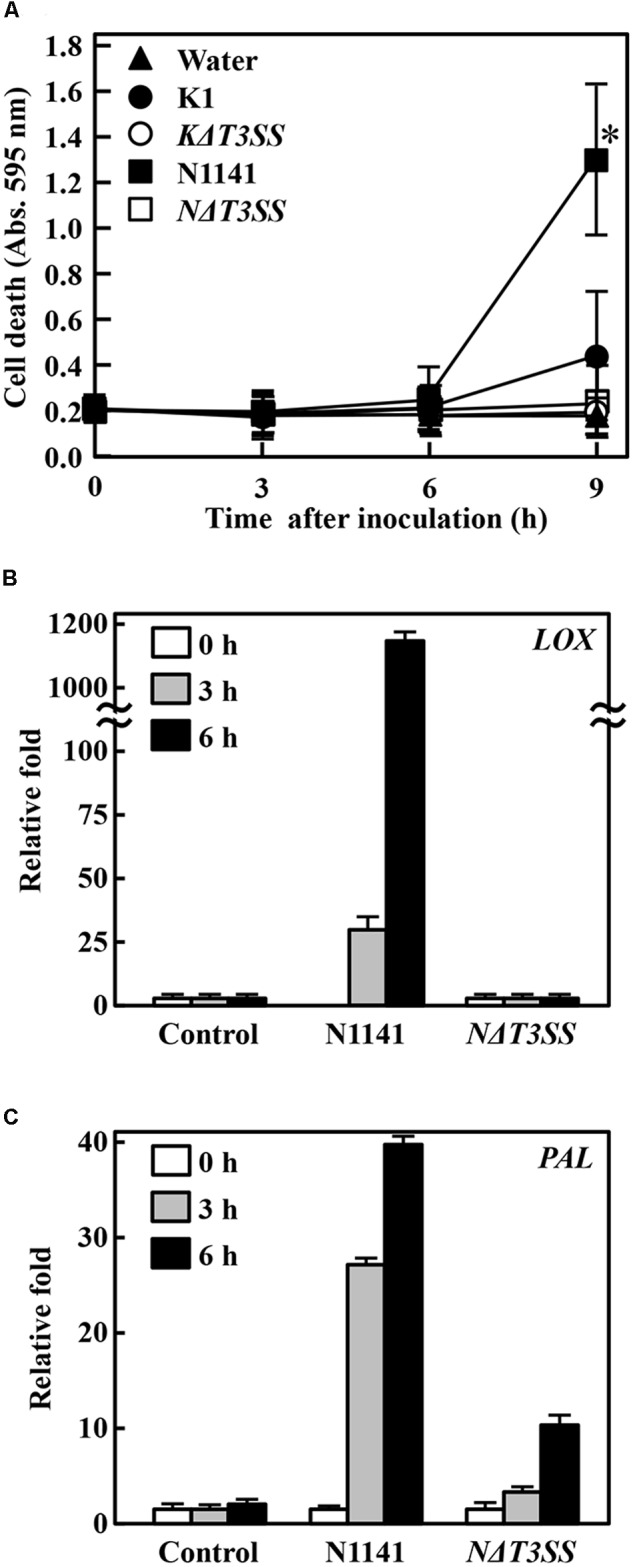
**Induction of effector-triggered immunity (ETI). (A)** Time course of HR cell death in cultured rice cells inoculated with K1 wild type (solid circles), *KΔT3SS* (open circles), N1141 wild type (solid squares), *NΔT3SS* (open squares), or water (solid triangle). HR cell death was detected by Evans Blue staining. Bars indicate standard deviation of three independent experiments. Asterisks indicated significant differences between N1141 and others according to *post hoc* ANOVA Tukey-Kramer test (*P* < 0.05). **(B)** *LOX* mRNA levels in cultured rice cells inoculated with N1141 or *NΔT3SS*. **(C)** *PAL* mRNA levels in cultured rice cells inoculated with N1141 or *NΔT3SS*. mRNA levels were calculated from the threshold point in the log-linear range of real-time RT-PCR. The *y-*axis represents the fold change in mRNA levels relative to those in cultured cells prior to treatment. Error bars indicate standard deviation of three experiments.

T3SS effector proteins often function as ETI induction factors. Therefore, we next used T3SS-deleted mutants to investigate whether N1141 and K1 possess T3SS effector proteins that induce ETI in non-host plants. When the rice-avirulent N1141 strain of *A. avenae* was inoculated into exponentially growing cultured rice cells, cell death detected by Evans Blue staining was observed 9 h after inoculation (**Figure 2A**). By contrast, *KΔT3SS* and *NΔT3SS* did not cause cell death in cultured rice cells until after 9 h of incubation, and a comparatively small amount of cell death was detected in K1-inoculated rice cells 9 h after incubation (**Figure 2A**). Mock-treated cultured cells and *KΔT3SS* exhibited no induction of cell death (**Figure 2A**). Because N1141, K1, *NΔT3SS*, and *KΔT3SS* grew at the same rate in culture media containing rice cells during this bioassay (Supplementary Figure S1), cell death might have been caused by effector proteins secreted through T3SS.

Phenylalanine ammonia lyase (PAL) catalyzes the deamination of L-phenylalanine to trans-cinnamic acid, the first step in the biosynthesis of lignin monomers and certain classes of phytoalexins (Zhu et al., 1995). Induction of lignin deposition in peripheral tissues and accumulation of furanocoumarin and isoflavonoid-derived phytoalexins help protect against pathogens (Lamb et al., 1989). Lipoxygenases (LOX) are a family of monomeric non-heme, non-sulfur/iron dioxygenases that catalyze the conversion of polyunsaturated fatty acids into conjugated hydroperoxides (Schaffrath et al., 2000; Maccarrone et al., 2001). LOX have also been proposed to form biologically active compounds during normal developmental stages, such as germination or growth, and as responses to environmental stress such as pathogen attack (Gardner, 1991; Véronési et al., 1996). We recently reported that expression of *PAL* and *LOX* genes are induced in cultured rice cells upon infection by N1141, but not K1, suggesting that *PAL* and *LOX* genes are upregulated during ETI (Tanaka et al., 2001). Therefore, to confirm that strain N1141 possesses ETI-inducible effector proteins, we used qRT-PCR to follow the time course of *PAL* and *LOX* gene expression activated by infection with N1141 or *NΔT3SS* in cultured rice cells. *PAL* and *LOX* transcripts were induced 3 h post-inoculation with N1141, and their levels gradually increased until 6 h (**Figures 2B,C**). By contrast, no induction of *PAL* and *LOX* genes was observed in *NΔT3SS*-inoculated rice cells (**Figures 2B,C**).

### Identification of T3SS Effector Candidates Controlling the Virulence of N1141 or K1

To identify the major determinant controlling the virulence of K1 or N1141 on host plants, we performed whole genome sequencing of both strains. The genome of *A. avenae* K1 consists of a circular chromosome of 5,387,858 bp with an average G + C content of 68.9% and a total of 5,138 CDSs. The genome of N1141 consists of a circular chromosome of 5,328,578 bp with an average G + C content of 68.7% and a total of 4,786 CDSs.

To identify T3SS effector candidates in the genome overall, we used EffectiveT3, a software package designed for amino acid sequence-based prediction of proteins secreted via the T3SS^1^ (Arnold et al., 2009). A total of 340 putative K1 proteins and 346 putative N1141 proteins were predicted to be secreted through T3SS. To determine whether these candidates included specific virulence factors, we performed an amino acid sequence homology analysis between the candidates from K1 and N1141. This comparison revealed that the putative protein encoded by Ngene_2122 had low sequence similarity to the putative protein encoded by Kgene_2283 (**Table 1**). Ngene_2122 encodes a protein with 17 LRR domains (Supplementary Figure S2). Accordingly, Ngene_2122 and Kgene_2283 were designated as N1141 Lrp and K1 Lrp, respectively.

**Table 1:** Comparative analysis of amino acid sequences between K1 and N1141 T3SS effectors.

Kl gene no.	N1141 gene no.	Identity (%)
Kgene_2283	Ngene_2122	25
Kgene_1334	Ngene_1263	33
Kgene_4233	Ngene_3954	36
Kgene_3906	Ngene_3655	38
Kgene_4943	Ngene_4612	38
Kgene_3445	Ngene_3237	39

*The corresponding gene numbers between K1 and N1141 were summarized in the table. Numbers indicated amino acid homology (%) between K1 and N1141 genes.*

### Identification of Lrp Protein as a Specific Virulence Factor

**FIGURE 3:**
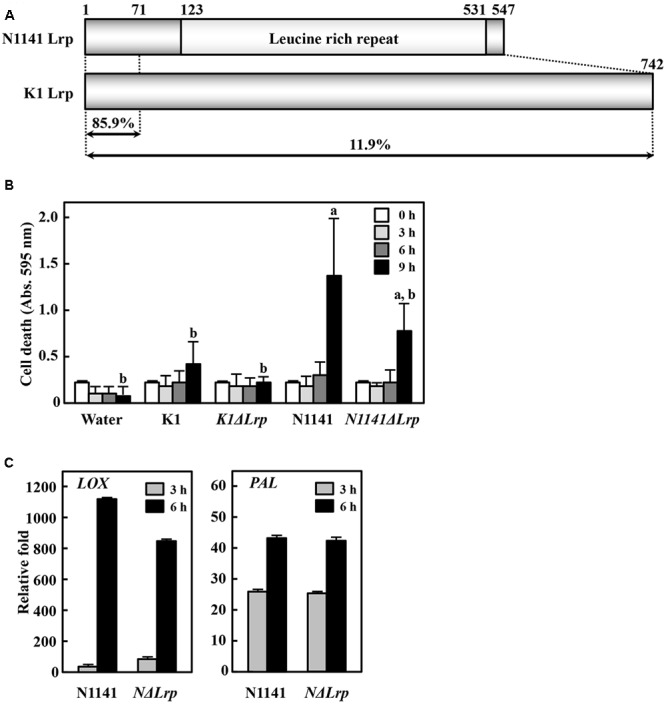
**Induction of immune responses in cultured rice cells by Lrp-deleted mutants. (A)** Structures of N1141 Lrp and K1 Lrp. Numerals on the box represent numbers of amino acids. **(B)** Induction of HR cell death in cultured rice cells inoculated with K1 wild type, *KΔLrp*, N1141 wild type, *NΔLrp*, and water. White, 0 h after inoculation; light gray, 3 h after inoculation; dark gray, 6 h after inoculation; black, 9 h after inoculation. Bars indicate standard deviation of three independent experiments. Small-case letters above bars indicate significant differences according to *post hoc* ANOVA Tukey-Kramer test (*P* < 0.05). **(C)** *LOX* (left figure) and *PAL* (right figure) mRNA levels in cultured rice cells inoculated with N1141 or *NΔLrp*. mRNA levels were calculated from the threshold point in the log-linear range of real time RT-PCR. The *y*-axis represents the fold change in mRNA levels relative to the levels in cultured cells before treatment. Bars indicate standard deviation of three experiments.

Although the nucleotide sequences of K1 *Lrp* and N1141 *Lrp* are very similar (94%), K1 Lrp contains no LRR motif due to a frame shift mutation resulting from a 1-bp insertion within K1 *Lrp* (Supplementary Figure S3). Given the low amino-acid sequence identity (25%) between K1 Lrp and N1141 Lrp, the proteins are likely to have different functions (**Figure 3A** and Supplementary Figure S4). To evaluate the functions of these proteins, we generated *Lrp*-deleted mutants in N1141 and K1 (*NΔLrp* and *KΔLrp*, respectively) by homologous recombination. In liquid medium, both mutants grew at the same rates as the corresponding wild-type strains (Supplementary Figure S1).

To elucidate the role of N1141 and K1 Lrp in ETI induction, we monitored HR cell death in cultured rice cells after inoculation with *KΔLrp* and *NΔLrp*. The same induction pattern of HR cell death was observed in cultured rice cells inoculated with *NΔLrp* and N1141 wild type (**Figure 3B**). By contrast, inoculation with *KΔLrp* or K1 wild type did not cause HR cell death until 9 h after inoculation, suggesting that neither N1141 Lrp nor K1 Lrp functions as an inducer of HR cell death in rice. Next, we monitored the time course of *PAL* and *LOX* gene expression in *NΔLrp*-inoculated rice. *PAL* and *LOX* transcripts were induced 3 h after inoculation with N1141, and gradually increased until 6 h (**Figure 3C**). When *NΔLrp* was inoculated into cultured rice cells, the same induction patterns of *PAL* and *LOX* were observed, indicating that the ETI of rice is induced by effector proteins other than N1141 Lrp.

**FIGURE 4:**
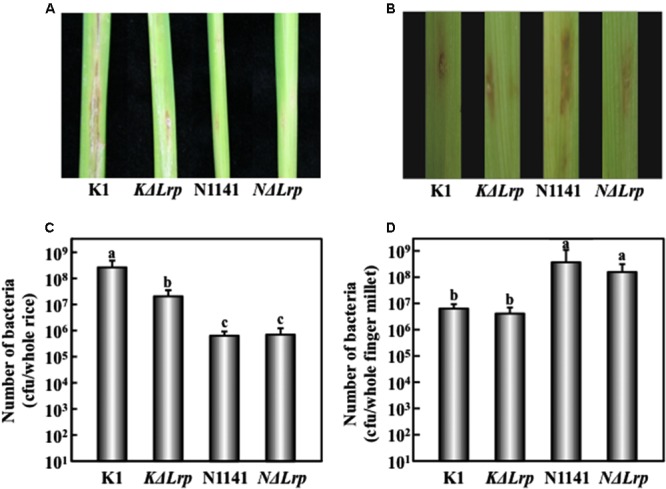
**Virulence test of Lrp-deleted mutants in rice and finger millet. (A)** Phenotype of brown stripe symptoms in rice 7 days after inoculation. **(B)** Phenotype of brown stripe symptoms in finger millet 7 days after inoculation. Bacterial strains (1 × 10^6^ cfu) were inoculated into 30-day-old rice seedlings using the single-needle method. **(C)** Number of bacterial cells in whole rice plants 4 days after inoculation. Bars indicate standard deviation of nine experiments. **(D)** Number of bacterial cells in whole finger millet plants 4 days after inoculation. Bars indicate standard deviation of nine experiments. Small-case letters above bars in **(C)** and **(D)** indicate significant differences according to *post hoc* ANOVA Tukey-Kramer test (*P* < 0.05).

To determine whether N1141 or K1 Lrp functions as a virulence factor in host cells, we performed inoculation tests. Brown stripe symptoms were observed in rice 7 days after K1 inoculation (**Figure 4A**). By contrast, inoculation of rice with the *KΔLrp* mutant produced milder symptoms (**Figure 4A**). When finger millet was inoculated with N1141 or the *NΔLrp* mutant, brown stripe symptoms were observed around the inoculation points 7 days later (**Figure 4B**). Milder brown stripe symptoms were observed when K1 and *KΔLrp* were inoculated into finger millet (**Figure 4B**).

We next tested for the presence of inoculated bacteria by measuring internal bacterial load at three time points. When K1 wild type (1 × 10^6^ cfu/μl) was inoculated into rice, the number of K1 cells reached 5 × 10^8^ cfu/plant 4 days after inoculation (**Figure 4C**). By contrast, no remarkable increase in the number of *KΔLrp* cells was observed 4 days after inoculation (**Figure 4C**). When tested on finger millet, N1141 and *NΔLrp* grew in the same manner, but no significant increase in the number of K1 or *KΔLrp* cells was observed (**Figure 4D**). These observations, together with the lesion formation data, indicate that the absence of K1 *Lrp* genes significantly decreased virulence to rice; however, unlike K1 Lrp, N1141 Lrp did not function as a virulence factor in finger millet.

### Identification of the Target Protein of K1 Lrp

**FIGURE 5:**
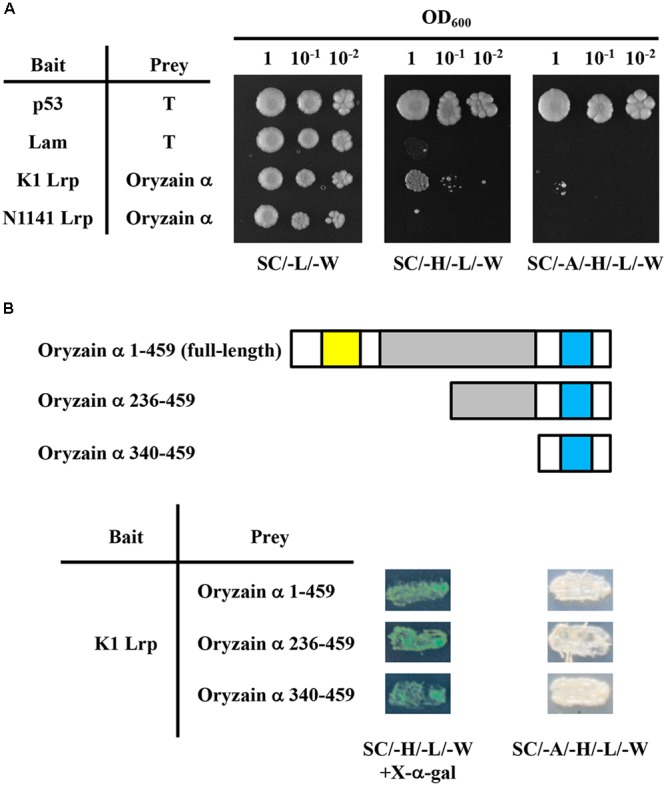
**Specific interaction between K1 Lrp and oryzain α in yeast. (A)** Test of interaction between K1 Lrp or N1141 Lrp and oryzain α using the yeast two-hybrid system. All transformants of yeast strain AH109 were serially diluted 10-fold and spotted on a synthetic complete medium and selection media. **(B)** Schematic representation of N-terminally truncated mutants of oryzain α and yeast two-hybrid analysis of their interactions with K1 Lrp. The yellow box represents the protease inhibitor domain (I29), the gray box represents the cysteine protease active domain (C1A), and the light blue box represents the protein interaction domain (granulin).

To investigate the virulence-related functions of K1 Lrp, we performed a yeast two-hybrid (Y2H) screen. For this purpose, we first constructed a cDNA library for Y2H screening. Total RNA was isolated from cultured rice cells infected with the N1141 or K1 strains of *A. avenae*. After purification of poly-A RNA, reverse transcription into cDNA was performed with random primers and oligo-dT primer, and cDNAs shorter than 500 bp were removed. A rice cDNA expression library was constructed by ligation of the size-fractionated and purified cDNAs into pGADT7 (*GADT7-cDNA*). To construct the bait vector, DNA encoding K1 Lrp was fused with pGBKT7 (*pGBKT7/K1 Lrp*). Approximately 6 × 10^5^ colonies were screened, and 36 colonies were selected as positive clones. Colony PCR was carried out on the positive colonies to confirm insertion of cDNA. After a careful sequence analysis of the PCR products, five abundant genes were designated *KLrpB1, KLrpB2, KLrpB3, KLrpB4*, and *KLrpB5* and 36 colonies were composed of 26 clones (*KLrpB1)*, four clones (*KLrpB2)*, four clones (*KLrpB3)*, one clone (*KLrpB4)*, and one clone (*KLrpB5)*. Among these genes, *KLrpB1* encoded oryzain α, which a papain-like cysteine protease. Many cysteine proteases function as anti-pathogen factors, suggesting that a specific interaction between K1Lrp and oryzain α decreases oryzain α function. Therefore, we next sought to determine whether the interaction between K1 Lrp and oryzain α is specific. To this end, full-length cDNAs of K1 and N1141 Lrp were amplified by PCR using specific primers and introduced into pGBKT7. K1 Lrp interacted strongly with oryzain α, whereas N1141 Lrp did not (**Figure 5A**).

Oryzain α contains three identified domains: a protease inhibitor domain (I29) in the N-terminus, a cysteine protease active domain (C1A) in the middle, and a protein–interacting domain (granulin) within the C-terminus. To identify the binding site of oryzain α, we produced deleted-bait vectors containing N-terminally truncated fragments of oryzain α. In Y2H analysis, all oryzain α fragments containing the granulin domain interacted with K1 Lrp, suggesting that K1 Lrp specifically interacts with the granulin domain of oryzain α (**Figure 5B**).

**FIGURE 6:**
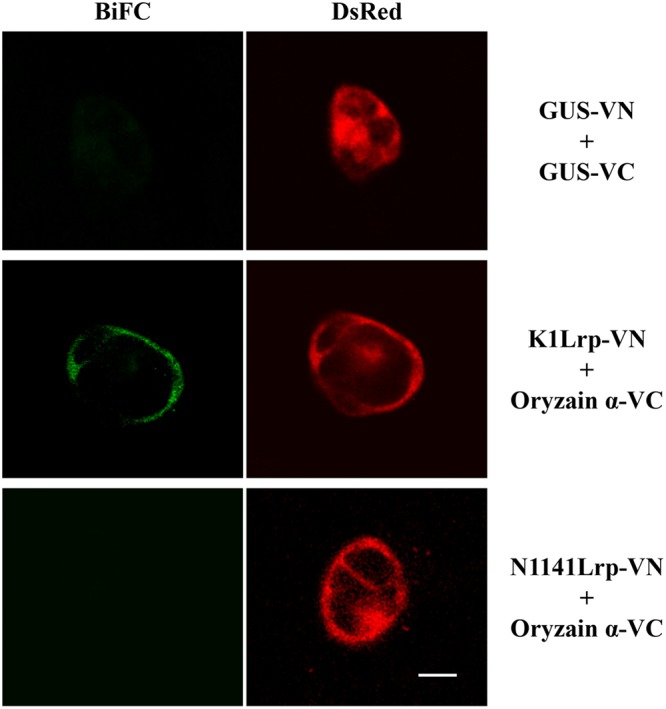
**Interaction between K1 Lrp and oryzain α detected by BiFC in cultured rice cells.** GUS-VN and GUS-VC (upper panels), K1Lrp-VN and Oryzain α-VC (middle panels), and N1141Lrp-VN and Oryzain α-VC (lower panels) were co-expressed in cultured rice cells, respectively. DsRed expression vector were co-transformed with BiFC vectors to identify the transformed cells. White bar showed 10 μm.

To confirm the interaction between K1 Lrp and oryzain α, K1 Lrp or N1141 Lrp were fused with the N-terminal fragment of Venus (K1Lrp-VN and N1141Lrp-VN), and the cDNA encoding oryzain α was fused with the C-terminal fragment of Venus (oryzain α-VC). When K1Lrp-VN or N1141Lrp-VN and oryzain α-VC were co-introduced into cultured rice cells, BiFC fluorescence was observed in the cytoplasm of cultured rice cells transformed with K1Lrp-VN and oryzain α-VC, whereas no fluorescence was observed in cultured rice cells transformed with N1141Lrp-VN and oryzain α-VC. These results indicate that K1 Lrp specifically interacts with oryzain α in rice cells (**Figure 6**).

## Discussion

Here, we provide experimental evidence that K1 Lrp is a virulent factor of *A. avenae* during infection of rice plants, whereas N1141 Lrp does not play a similar role in finger millet. Lrp of *A. avenae* rice avirulent N1141 strain which is novel a leucine-rice repeat protein shares high sequence identity with the GALA7 protein from *Ralstonia solanacearum* (Angot et al., 2006). GALA7 specifically interacts with its target, the SCF-type E3 ubiquitin ligase protein complex, and functions as virulence factor. Interestingly, deletion of *GALA7* does not affect *R. solanacearum* virulence on *Arabidopsis*. This could be because GALA7 protein is a virulence determinant, but functionally redundant with other GALAs, of which at least seven are encoded by the *R. solanacearum* genome (Kajava et al., 2008). Whole-genome analysis revealed that N1141 possesses several homologs of Lrp. The fact that a single Lrp mutant of N1141 did not affect virulence in the host plant (finger millet) and in a non-host plant (rice) could be explained by redundancy between Lrp and other homologs, even though N1141 Lrp still functions as a virulence factor. In future work, a multiple mutant lacking both Lrp and its homologs should be used to clarify whether the N1141 Lrp acts as a virulence factor in host or non-host plants.

Although the nucleotide sequences of K1 and N1141 *Lrp* genes were very similar, K1 Lrp has no LRR motif in the predicted amino acid sequence. This is due to a frame shift mutation resulting from a 1-bp insertion within K1 Lrp. The fact that the amino acid sequence of K1 Lrp is very different from that of N1141 Lrp raises questions about whether K1 Lrp functions as a T3SS effector protein. To address this question, K1 Lrp and N1141 Lrp were evaluated as putative secretory proteins using EffectiveT3, a software package designed for sequence-based prediction of secreted proteins^1^. The secretion analysis predicted that both proteins are secreted via T3SS. These results indicate that K1 and N1141 Lrp proteins both function as effectors despite their low amino-acid sequence identity. The N-terminal regions of both proteins are very similar, suggesting that these domains are responsible for secretion through the T3SS. Thus, frame shift via 1-bp insertion might be a rational method for acquiring function as a virulence factor.

Y2H screen using the K1 and N1141 Lrp proteins as bait revealed that K1 Lrp interacts with oryzain α, whereas N1141 Lrp does not. The interaction also was observed in BiFC assay with cultured rice cells. Rice oryzain α is a member of the pathogenesis-related protein (PR) 7 family (van Loon et al., 2006). Most PR proteins exhibit direct antimicrobial activities, such as chitinase and β-1, 3-glucanase, that degrade chitin and glucan, respectively, whereas others are proteases that hydrolyze pathogenic proteins (Mestre et al., 2016). PR proteins play roles in both constitutive and induced defense responses. For instance, several plants such as potato and tomato contain basal levels of proteases in their apoplasts, including serine protease–like P69 and cysteine protease–like Rcr3, which are required for tomato resistance against *Cladosporium fulvum* (Song et al., 2009), and *Phytophthora* inhibited protease 1 (Pip1), a potato resistance factor to *Phytophthora infestans* (Ilyas et al., 2015). In addition to their functions as constitutive defense factors, several PR-containing proteases are induced both locally and systemically after pathogen infection, suggesting that their activities directly or indirectly affect pathogen growth. Deletion or silencing of genes encoding proteases increases the susceptibility of plants to pathogens, supporting the idea that these genes play roles in defense responses. Deletion of Rcr3 increases the susceptibility of tomato to *P. infestans* and *C. fulvum*. Likewise, silencing of C14 in *Nicotiana benthamiana* significantly increases susceptibility to *P. infestans* (Ilyas et al., 2015). These data indicate that proteases play a determinative role in the execution of defenses against plant pathogens. Accordingly, a promising strategy for pathogens would be to evolve effectors that can disable PR proteins containing cysteine protease domains. Thus, the interaction of the K1 Lrp protein with oryzain α might increase the susceptibility of rice to *A. avenae* K1 by disabling oryzain α.

Truncation experiments using oryzain α revealed that K1 Lrp interacts specifically with the granulin domain of oryzain α. The granulin domain was originally identified in a number of small mammalian proteins called granulins and epithelins, which were thought to be regulators of cell growth. The cysteine-rich protein repeats resembling granulin sequences are also present in the C-termini of several inducible plant cysteine proteases. Their common occurrence as C-terminal extensions of a variety of plant cysteine proteases suggests that they play conserved roles in regulating the activities of these enzymes (Tolkatchev et al., 2001). To clarify the role of granulin domains within plant cysteine proteases, a 35–amino acid residue peptide corresponding to the N-terminal subdomain of the granulin domain from oryzain β (a homolog of oryzain α) was synthesized, and the tertiary topology in solution was studied using NMR. The tertiary topology of the resultant peptide consisted of a stack of two β-hairpins. The β-hairpin stack, a novel protein architecture first found in carp granulin-1, is arranged in two ladders of four β-strands zipped together by seven reverse turns and two symmetrical arrays of interacting disulfide bonds (Tolkatchev et al., 2001). This structure is thought to play a significant role in specific protein–protein interactions. The binding of K1 Lrp to the granulin domain might inhibit the function of oryzain α. Further three-dimensional structural analysis will be necessary to determine whether K1 Lrp binds the β-hairpin stack of oryzain α.

## Author Contributions

MK, YY, and F-SC conceived and designed the experiments. MK, HH, YY, AS, and TK performed the experiments. MK and HH analyzed the data. MK and TF performed computational analysis. MK, HH, TF, and F-SC wrote the paper. All authors read and approved the final manuscript.

## Conflict of Interest Statement

The authors declare that the research was conducted in the absence of any commercial or financial relationships that could be construed as a potential conflict of interest.

## Footnotes

1 http://www.effectivedb.org/effective/submit
