# Supplementary material for: Frameshift Mutation Confers Function as Virulence Factor to Leucine-Rich Repeat Protein from Acidovorax avenae
Source: Front Plant Sci. 2017 Jan 4;7:1988. doi: 10.3389/fpls.2016.01988 (PMC5209373; doi:10.3389/fpls.2016.01988)
Supplement: Supplementary file 1 [file Table_1.pdf]

Supplemental Table S1 | Primers used in this article.

Name of primer	Sequence (5' to 3')
**PAL gene-F**	**GGCGAGGAGTGCAACAAGGTGTT**
**PAL gene-R**	**TGGGTGTATGGCAATGGCAATGG**
***LOX* gene-F**	**CAGACGAGGCCTGGAACAGCGA**
***LOX* gene-R**	**TTCATCAGCTGGTACGGCAGGAT**
**K1-29231-F-up_spe**	**ACTAGTCTCTTCATCTGTATGGCCGCC**
**K1-30297-R-up**	**GGCTCGATCGATCGATCGCTCGAAGCGGATCGCCTGTTCGATTT**
**K1-32115-F-down**	**TTCGAGCGATCGATCGATCGAGCCTGCCATCTCTCTTTCGGACTGG**
**K1-33096-R-down_spe**	**ACTAGTCGATGACCAGCTCGACGATGA**
**KLRP-UP-F**	**GTCTGCGAGGTGGAGGCACCG**
**KLRP-UP-R**	**GGGCCTTGTCGGACATCGCGCTGATCCGACGGTC**
**KLRP-DOWN-F**	**CGCGATGTCCGACAAGGCCCTCCACAGCCCCT**
**KLRP-DOWN-R**	**GTCGACGCAACTGGTCACGATGCT**
**K1 Lrp (BKT7-EcoRI)-F**	**GAATTCATGTCCGCGCCGCCGCAG**
**K1 Lrp (BKT7-BamHI)-R**	**GGATCCTCAGCCATCGCCAACCTGGAGTTGTC**
**N1141 Lrp (BKT7-EcoRI)-F**	**GAATTCATGTCCGCACCGCCGCAGAC**
**N1141 Lrp (BKT7-EcoRI)-R**	**GAATTCTCAGCCGGCCGCCCGG**
**Oryzain(EcoRI)-F**	**GAATTCATGAGGATTTCCATGGCTCTCG**
**Oryzain(EcoRI)-R**	**GAATTCTCAAGCGCTGCTCTTCTTGC**
**Oryzain 236 (EcoRI)-F**	**GAATTCACAATTGACAGCTACGAAGATGTAA**
**Oryzain 340 (EcoRI)-F**	**GAATTCCCATCCTACCCTCTGAAGAAGG**
**N1141_lrp_pENTER_cacc_F**	**CACCATGTCCGCACCGCCGCAGACCA**
**N1141_lrp_pENTER_without_stop_R**	**GCCGGCCGCCCGGCTCAC**
**K1_lrp_pENTER_cacc_F**	**CACCATGTCCGCGCCGCCGCAG**
**K1_lrp_pENTER_without_stop_R**	**GCCATCGCCAACCTGGAGTTGTCC**
**oryzain_pENTER_cacc_F**	**CACCATGAGGATTTCCATGGCTCTCG**
**oryzain_pENTER_without_stop_R**	**AGCGCTGCTCTTCTTGCC**
**GUS-pENTR_F**	**CACCATGTTACGTCCTGTAGAAAC**
**GUS-pENTR_R**	**TTGTTTGCCTCCCTGCTGC**
