# Supplementary figures and images for: Frameshift Mutation Confers Function as Virulence Factor to Leucine-Rich Repeat Protein from Acidovorax avenae
Source: Front Plant Sci. 2017 Jan 4;7:1988. doi: 10.3389/fpls.2016.01988 (PMC5209373; doi:10.3389/fpls.2016.01988)

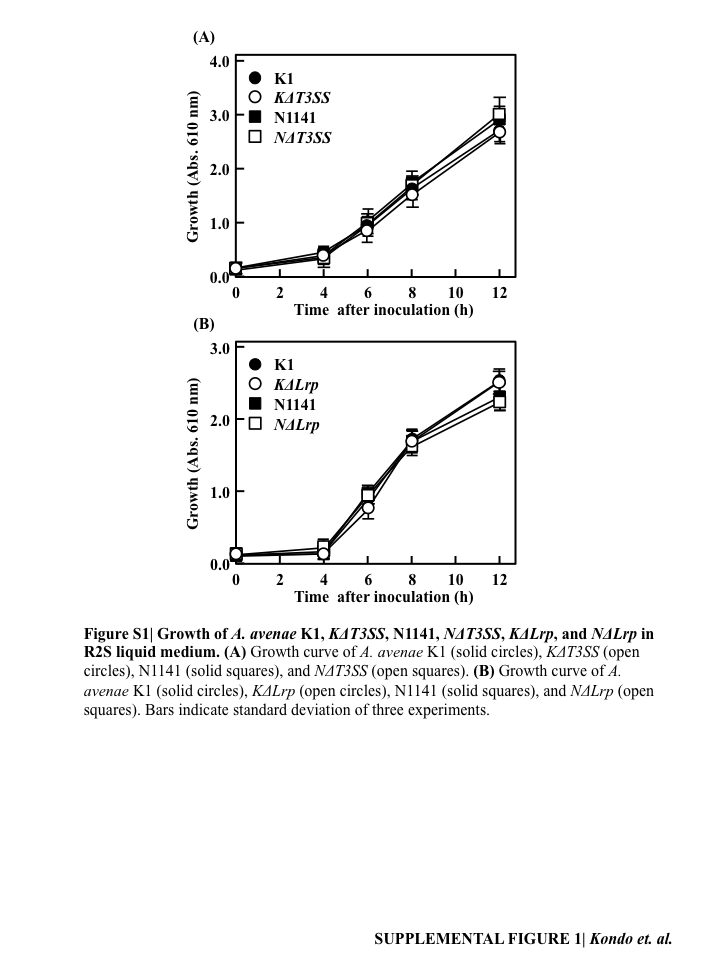

Supplement: Supplementary file 2 [file Image_1.tiff]

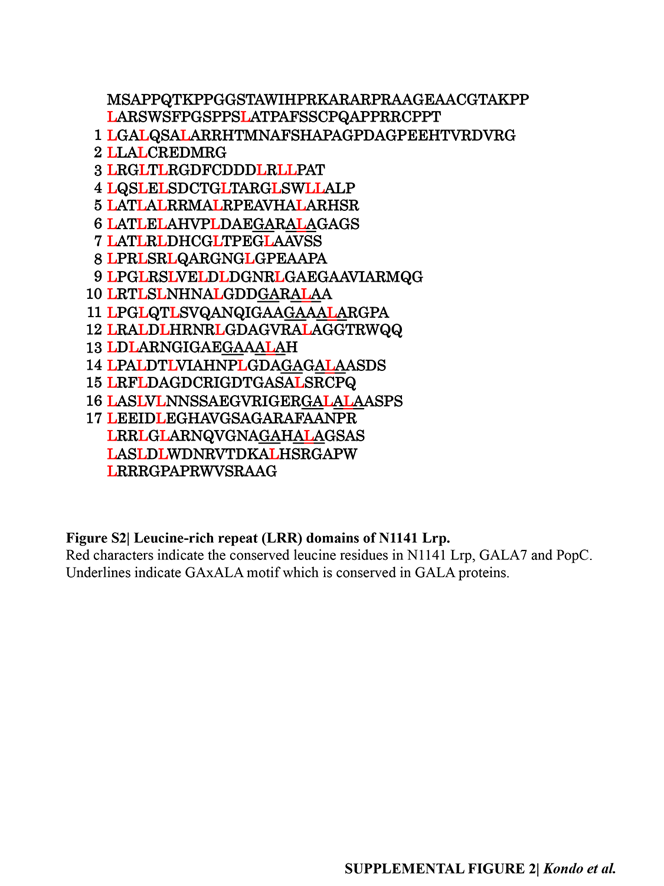

Supplement: Supplementary file 3 [file Image_2.TIF]

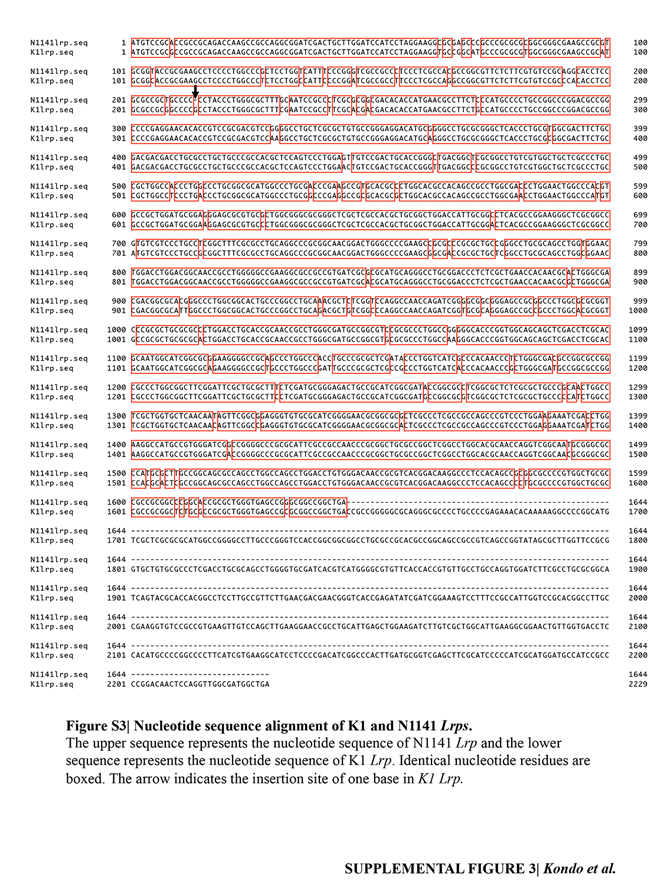

Supplement: Supplementary file 4 [file Image_3.TIF]

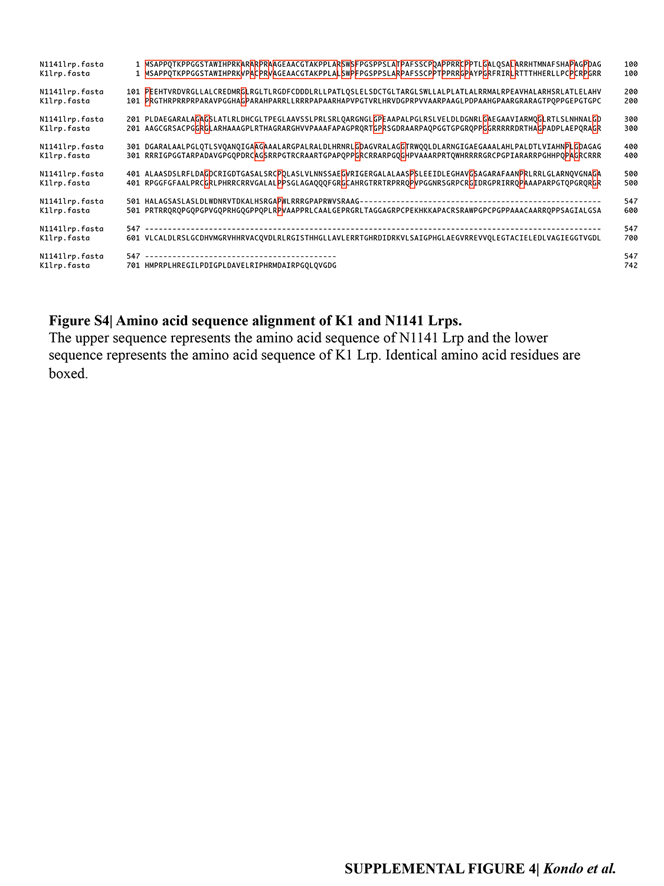

Supplement: Supplementary file 5 [file Image_4.TIF]
